# Insights from EEG analysis of evoked memory recalls using deep learning for emotion charting

**DOI:** 10.1038/s41598-024-61832-7

**Published:** 2024-07-24

**Authors:** Muhammad Najam Dar, Muhammad Usman Akram, Ahmad Rauf Subhani, Sajid Gul Khawaja, Constantino Carlos Reyes-Aldasoro, Sarah Gul

**Affiliations:** 1 National University of Sciences and Technology, Islamabad, 44000 Pakistan; 2 Department of Computer Science, School of Science and Technology, City, University of London, Northampton Square, London, EC1V 0HB UK; 3 Department of Biological Sciences, FBAS, International Islamic University, Islamabad, Pakistan

**Keywords:** Emotional memory recall, Electroencephalogram (EEG), Ultra-mobile wearable sensor, Memory-induced emotion recognition, Affective words, Biomedical engineering, Emotion

## Abstract

Affect recognition in a real-world, less constrained environment is the principal prerequisite of the industrial-level usefulness of this technology. Monitoring the psychological profile using smart, wearable electroencephalogram (EEG) sensors during daily activities without external stimuli, such as memory-induced emotions, is a challenging research gap in emotion recognition. This paper proposed a deep learning framework for improved memory-induced emotion recognition leveraging a combination of 1D-CNN and LSTM as feature extractors integrated with an Extreme Learning Machine (ELM) classifier. The proposed deep learning architecture, combined with the EEG preprocessing, such as the removal of the average baseline signal from each sample and extraction of EEG rhythms (delta, theta, alpha, beta, and gamma), aims to capture repetitive and continuous patterns for memory-induced emotion recognition, underexplored with deep learning techniques. This work has analyzed EEG signals using a wearable, ultra-mobile sports cap while recalling autobiographical emotional memories evoked by affect-denoting words, with self-annotation on the scale of valence and arousal. With extensive experimentation using the same dataset, the proposed framework empirically outperforms existing techniques for the emerging area of memory-induced emotion recognition with an accuracy of 65.6%. The EEG rhythms analysis, such as delta, theta, alpha, beta, and gamma, achieved 65.5%, 52.1%, 65.1%, 64.6%, and 65.0% accuracies for classification with four quadrants of valence and arousal. These results underscore the significant advancement achieved by our proposed method for the real-world environment of memory-induced emotion recognition.

## Introduction

Emotions are shaped not only by immediate stimuli but also by past experiences and memories. In the real-world environment, memories can induce emotions in the absence or minimal presence of external stimuli. Humans usually recall their emotional memories for emotional regulation in real-world scenarios by repeatedly feeling those emotional states^1^. However, the research on automatic emotion recognition predominantly relies on immediate stimuli for emotion elicitation. Therefore, the dataset acquisition is generally constrained to a specific lab environment and the presence of external stimuli, such as horror or comedy movies, to induce immediate emotional responses in the participants. Because of the limitations posed by immediate stimuli, automatic emotion recognition algorithms usually fail to perform well in real-world scenarios. Emerging research works^2,3^ with self-induced or emotional memories instead of immediate stimuli to play a crucial role in understanding and recognizing emotions accurately in real-world applications. Therefore, developing and improving techniques that facilitate the generation of emotional memories could be highly effective for the industrial-level usefulness of automatic emotion recognition.

The motivation for memory recall-based emotion analysis originates from several studies highlighting the interplay between emotions and memory. The strong correlation between inducing emotional responses through stimulus images and subsequent memory recall demonstrates the relevance of memory formation and emotional stimuli^4^. Their study was limited to static instead of interactive user experience and is limited to 37 university students with a limited age range of 18–29 years. Another research^5^ found that memories triggered with autobiographical images of favorite places can effectively induce positive emotions, particularly useful for depression patients. Despite indicating the effectiveness of autobiographical memories in inducing emotions, they do not provide automatic emotion recognition for these memory-induced emotions, and the study has limited generalizability because of specific age groups of participants (between 18 and 35 years and above 65 years). The researchers^6^ also explored the association between emotional states and false memories, revealing that false memories can occur more frequently in the context of positive emotions. Another study^7^ highlights the influence of positive and negative body postures on EEG patterns during emotional memory recalls. Similarly, the analysis^8^ provides chaotic EEG patterns during the recall of fearful events in memory. These studies provide insight into memory-induced emotion phenomena and potential implications for emotion recognition.

There are two models for emotion charting: the categorical model and the dimensional model. The categorical model includes various emotion categories such as happy, sad, fear, disgust, surprise, and anger. The dimensional model is based on the valence and arousal on the scale of integer values. The valence is the measure of pleasure or displeasure, while arousal is the measure of excitement. The prevailing trend in EEG-based emotion recognition primarily focused on the binary classification of high and low levels of either valence or arousal, particularly with challenging self-induced and memory-induced emotions^9^. However, it may oversimplify the diverse spectrum of human emotions. Despite the potential benefits of considering all four quadrants of valence and arousal, only a limited subset of studies^9^ utilized this comprehensive framework. This underscores the need to explore all quadrants of valence and arousal, mitigating potential criticism and ensuring a robust understanding of affective computing.

Memory-induced emotion recognition is explored with conventional machine-learning techniques. An earlier study^10^ proposed the real-time identification of self-induced disgust by remembering unpleasant odors using electroencephalogram (EEG) signals. Their study lacks exploration of a broader range of emotions and is limited to a small sample size (ten subjects only). Another study^11^ with a relatively larger dataset of 28 subjects was also limited to disgust emotion, as the EEG correlates with odor memory, even when the person affected by hyposmia imagines an olfactory situation (disgust). They find that the subjects can lose concentration during memory recall, which affects emotion recognition performance.

Deep learning is the most commonly used recent tool for improved emotion recognition performance, but this tool is underexplored for memory-induced emotion recognition due to a lack of relevant data. Conventional techniques were limited to perform with a small sample size and number of emotion classes as they can extract a limited set of features from EEG signals (spatial or temporal features). A study^12^ explored regularization parameter-based improved intrinsic feature extraction method for EEG signals via empirical mode decomposition (EMD) to effectively enhance depression recognition performance on four EEG datasets. In recent years, EEG signal analysis for emotion recognition has enhanced our understanding of neural correlates and improved classification accuracy and robustness by leveraging deep learning algorithms with diverse features. A recent study^13^ used an improved capsule network and residual Long-Short Term Memory (ResLSTM), and another study used multi-branch Capsule network^14^ to extract spatiotemporal dual module features for improving emotion recognition performance. The combination of CNN and LSTM is explored for better emotion recognition performance^15^. Few researchers^16,17^ also explored 1D-CNN for EEG signal analysis for emotion recognition, as it can extract repetitive and unique patterns from 1D channel data of EEG signals. However, these studies lack the testing on self-induced or memory-induced-based challenging datasets. The existing work has primarily explored emotions induced with immediate external stimuli, but the potential of deep learning in extracting neural signatures of internally induced or memory-induced emotions remains largely untapped^18^. This paper aims to bridge this gap by presenting a novel deep learning-based approach associated with memory-induced emotions evoked by affective words using EEG analysis.

The current challenges in EEG-based emotion analysis are the restriction of natural emotional expression due to the requirement of remaining still to avoid movement artifacts during EEG acquisition, the scarcity of research applying self-designed models to real-world applications instead of pre-trained models, and the significant effect of choice of k-value in cross-validation for model’s generalization ability, particularly with non-random splits and small datasets, lead to overfitting and artificially inflated accuracy rates^19^. Existing studies utilize auditory and visual stimuli to evoke memory-induced emotions, including affective words^20,21^. The affective words have an inherent ability to evoke personalized semantic association and mental imagery and are more versatile to subjective experiences compared to images and audio. Despite the advancement in EEG-based emotion recognition research, the state-of-the-art requirements include flexibility of EEG acquisition, custom deep learning models suitable for EEG signal data, more rigorous evaluation of model performance with leave-one-out validation, adaptability of deep learning models for memory-induced emotions, selection of stimuli to evoke memory-induced emotions, and detailed performance metrics such as accuracy, sensitivity, specificity, and F-measure.

Memory recall-based systems are emerging and challenging for emotion recognition. From existing research on emotion recognition, it is evident that emotional memory recall-based systems are never explored with deep learning frameworks to improve the performance of emotion recognition and are also not explored with emotional memories induced by affective words. Therefore, the affective words to use stimulus for emotional memories contributed to the novel dataset. The specific work of this paper includes a dataset to evoke emotional memories with affective words, and the use of novel deep learning framework for improved recognition performance. In a real-world environment, it is significant to acquire the emotional profile of persons, while they are busy with daily activities and think freely about any autobiographical emotional memory. The major contribution of this research is summarized as follows.

1. This research improved recognition performance for the real-world environment of highly subjective memory-induced emotion with triggering words and a large population size.
2. One dimensional convolutional neural network followed by the recurrent neural network referred to as 1D convolutional recurrent neural network (1D-CRNN) is proposed as a feature extractor with an extreme learning machine (ELM) as a classifier for emotion recognition with an ultra-mobile EEG cap.

The remaining part of this article is divided into background literature, dataset acquisition, methodology, results, and discussion. The last section then concludes the article with conclusive remarks.

## Background

Learning, memory retention, and recall are primary cognitive functions of the human brain. There are two types of memories, long-term and short-term memory. Both types have different mechanisms to hold and retrieve memory content from the human brain. The physiology of memory recall is reviewed by^1^, investigating the interaction of brain regions during memory recall tasks using EEG signals. The prefrontal cortex region of the brain, associated hippocampus cortices, and their interaction with other lobes are responsible for emotional memory recall. Various brain regions are associated with different types of memories. For instance, visual memory links with the occipital lobe, episodic memory links with the mammillary body, spatial memory links with the parietal lobe, and short-term memories associated with the hippocampus and frontal lobe. Hippocampus also plays a role in memory management by moving the short-term memory to long-term memory. The exciting and emotional memories are associated with the amygdala part of the brain. The findings encourage the emotion recognition process during the natural phase of memory recalls.

**Table 1:** State-of-the-art machine learning techniques for memory-induced emotion recognition using EEG signals with dataset information, compared with proposed technique and dataset.

Study	Method	Evoked memory technique	Modality	Classes	Subjects
Chanel et al.^22^	Temporal and frequency domain features with Linear discriminant analysis (LDA) classifier	Memory recall relevant to personalized stimulus images	EEG (62 channels)	Three classes (positive, negative, neutral)	10
Iacoviello et al.^23^	Wavelet transform feature extraction, Principal component analysis for feature selection, and SVM for classification	Memory recall of unpleasant odors	EEG (8 channels)	Two classes (Disgust or not disgust)	10
Zhuang et al.^24^	Differential entropy features, and SVM for classification	Memory recall of recently displayed video stimulus	EEG (62 channels)	Six basic emotions	30
Proposed	One dimensional convolutional recurrent neural network with combination of extreme learning machine (1D-CRNN-ELM)	Memory recall with displayed words	EEG (14 channels)	Four emotion classes (HVHA, HVLA, LVHA, LVLA)	69

### Conventional techniques for memory-induced emotion recognition

The memory-induced emotions are studied using conventional machine learning techniques. An earlier attempt^22^ proposed a combination of support vector machines and linear discriminant analysis to classify three emotions (positive, negative, and neutral) from memory recall. The emotions were induced by displaying relevant images for 8 seconds and then asking the users to recall relevant memories. EEG data recording utilizes the Biosemi Active II system, with 64 electrodes positioned according to the 10-10 system. The authors report the 63% classification accuracy for the three positive, negative, and neutral states of emotion. Another study^10^ utilized conventional strategies such as wavelet transform, principal component analysis (PCA), and support vector machine (SVM) to achieve 90% accuracy for the simple binary emotion classification problem, the presence of disgust or not. Another study^11^ also achieved similar results (90%) for the binary classification of either disgust or not through remembering unpleasant odor, but for a relatively larger dataset size of 28 subjects. The comprehensive description of the state-of-the-art techniques used for memory-induced emotion recognition using EEG signals is provided in Table 1.

**Table 2:** Comparison of state-of-the-art emotion databases using physiological signals.

Dataset	AMIGOS^25^	DEAP^26^	DECAF^27^	DREAMER^28^	MAHNOB-HCI^29^	Imagined Emotions^30^	MEMO
Participants	40 (27M, 13F)	32 (16M, 16F)	30 (16M, 14F)	23 (14M, 9F)	30 (13M, 17F)	32 (13M, 19F)	69 (36M, 33F)
Modalities	EEG, ECG, GSR and audio-visual	EEG, GSR and peripheral signals	ECG and peripheral signals	EEG, ECG	EEG, ECG, GSR and peripheral signals	EEG, ECG, EMG	EEG
Self-assessment annotations	Dimensional: valence, arousal, dominance, liking, familiarity. Categorical: Six basic emotions.	Dimensional: arousal, valence, liking, dominance and familiarity.	Dimensional: valence, arousal and dominance.	Dimensional: Valence, arousal and dominance.	Dimensional: valence, dominance.	Categorical: Love, joy, anger, fear etc.	Dimensional: valence, arousal.
Dimensional scale	1 to 9	Continuous scale 1 to 9	0 to 5 and − 2 to + 2	1 to 5	1 to 9	Non metric multi-dimensional scale	− 4 to 4
Acquisition	14 Channel EEG, Wireless ECG and GSR	32 Channel EEG and wired GSR	3 channel ECG	14 Channel EEG, Wireless ECG	32 Channel EEG and wired ECG, GSR	256-Channel Biosemi wired	64-channel Wireless sports cap (ANT Neuro)^31^
Age (years)	21–40 ($\mu$ = 28.3)	19–37 ($\mu$ = 26.9)	($\mu$ = 27.3, $\sigma$= 4.3)	22–33 ($\mu$= 26.6, $\sigma$ = 2.7)	19–40 ($\mu$ = 26.06, $\sigma$ = 4.93)	18–38 ($\mu$= 25.5, $\sigma$= 5)	20–56 ($\mu$= 36.95, $\sigma$= 9.67)
Stimuli	20 Videos	40 Videos	32 Videos	18 Videos	20 Videos	15 Sounds	16 words Memory recall

M represented male, F represent female, $\mu$ represents mean and $\sigma$ represents standard deviation.

A recent study^32^ investigates EEG and ECG analysis for emotional memory recall with audio-visual stimuli provided in three repetitions. The participants watched movies for 40 seconds and then closed their eyes for 180 seconds to recall those videos, providing self-assessments on the scale of valence and arousal. The primary finding of the research is a delayed response from ECG compared to EEG for pleasant memories compared to a simultaneous response of EEG and ECG for unpleasant memories. The study by^33^ highlights the significance of EEG frequency bands (delta, theta, alpha, beta, and gamma) and each brain region (all electrodes) in the emotional memory recall process. They examined EEG-based brain region activity across positive, negative, and neutral emotional states during memory recall of words and numbers. In another study^24^, the binary and six class classifications of partially memory-induced emotions (remembering recently experienced movie-induced emotions) are analyzed with EEG signals. The authors report the binary classification of positive emotion with 87.36% and negative emotion with 54.52% accuracy for six emotion classes. This study did not incorporate multi-class classification and subjective emotional memory recalls and was based on working memory of audio-visual stimuli.

### Limitations of existing datasets for memory-induced emotions

The existing datasets of emotion recognition such as AMIGOS^25^, DEAP^26^, DECAF^27^, DREAMER^28^, MAHNOB-HCI^29^ predominantly focus on stimuli-induced emotions rather than emotions evoked by memory recalls as presented in Table 2. These datasets use visual stimuli to induce emotions and are limited by small sample sizes, acquisition without mobility, and the narrow age range of participants. Emotion elicitation through stimulus videos is common practice in emotion recognition research, but the natural scenarios are quite different. To mimic the natural scenarios, a study^34^ focuses on the recollection of emotional experiences because these are the reflection of real-world experiences rather than simple reactions to specific stimuli, showing the significance of memory-induced emotion compared to immediate stimuli in future works.

In 2021, a study^35^ investigates a novel procedure to self-induce memories and recorded facial expressions for emotion recognition. The positive, negative, and neutral memory recalls are evoked using two mechanisms. The first mechanism includes semi-structured interviews created by expert researchers, and the second mechanism involves guided recalls through listening to statements related to interviews conducted five days earlier. Their study lacks the empirical analysis of emotion classification performance based on their algorithm. A dataset named **Imagined Emotions**^30^ is also available reflecting the correspondence between real feel emotions and recalled emotions in an autobiographic way. It aimed to recall emotions evoked by audio stimuli and then imagine the emotional scenario of recalling the situation. However, the dataset has a limited age range of 18-38 years from only 32 subjects.

With minimal evoking memory recall, the participant can think about any memory, either pleasant or unpleasant. A study^36^ of physiological responses investigates the relationship between change in EEG signal during free recall of words for different time-scale attention and success or failure of the recalled word. The main findings are the higher P300 amplitudes of EEG signals for the recalled words compared to the failure of the recall. This study does not incorporate any emotions but suggests the significance of emotional words in evoking free memory recalls. However, no available dataset incorporates minimal evoking external stimuli, such as affective words, to trigger memory-induced emotions. The dataset with minimal evoking stimuli is required for eliciting genuine emotional responses reflective of real-life experiences. Therefore, this study collected the EEG dataset with memory-induced emotions evoked by affective words. A comparison of our collected dataset is compared against some popular datasets such as AMIGOS^25^, DEAP^26^, DECAF^27^, DREAMER^28^, MAHNOB-HCI^29^, and Imagined Emotions^30^ is presented in Table 2.

### Research gaps

Despite the significance of emotional memory recalls, the existing literature depicts several research gaps. The existing literature lacks the exploration of deep learning to improve memory-induced emotion recognition. The majority of studies used conventional machine learning techniques, and are limited to binary emotion classification, and results with small sample sizes. The literature also lacks the EEG dataset with subjective memory recalls with minimally evoking emotion. Memory recall from words is more subjective and oriented towards real-world scenarios than memory recall from images or audio-visual stimuli. This study addressed the challenges by proposing a 1D-CRNN-ELM framework and acquiring a dataset by displaying affective words and then asking the participants to recall any autobiographical memory related to that word, either positive or negative.

## Material and methods

### Dataset acquisition

The collected data was part of a large research study investigating the effect of stress on the brain and emotions. This dataset is useful in clinical settings, with various cognitive syndromes related to emotional memory. The authors assert that all procedures contributing to this work conform to the Malaysian Guideline for Good Clinical Practice (MGGCP), the ethical standards of the institutional committee on human experimentation, and the Declaration of Helsinki (1975), as revised in 2008. All trials involving human subjects were approved by the Medical Ethics and Research Committee of Prince Court Medical Centre, Malaysia.

**Figure 1:**
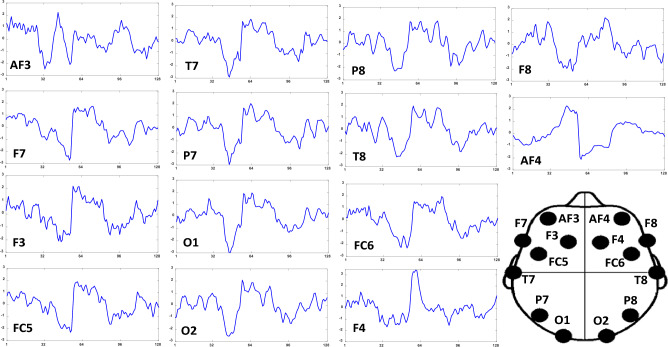
One of the sample of HVHA class of 1-sec EEG data, this figure illustrate the first segment plot (with sampling frequency of 128 Hz) of all the 14 channels according to 10–20 international standard^40^ of electrode placement incorporated in our study.

**Figure 2:**
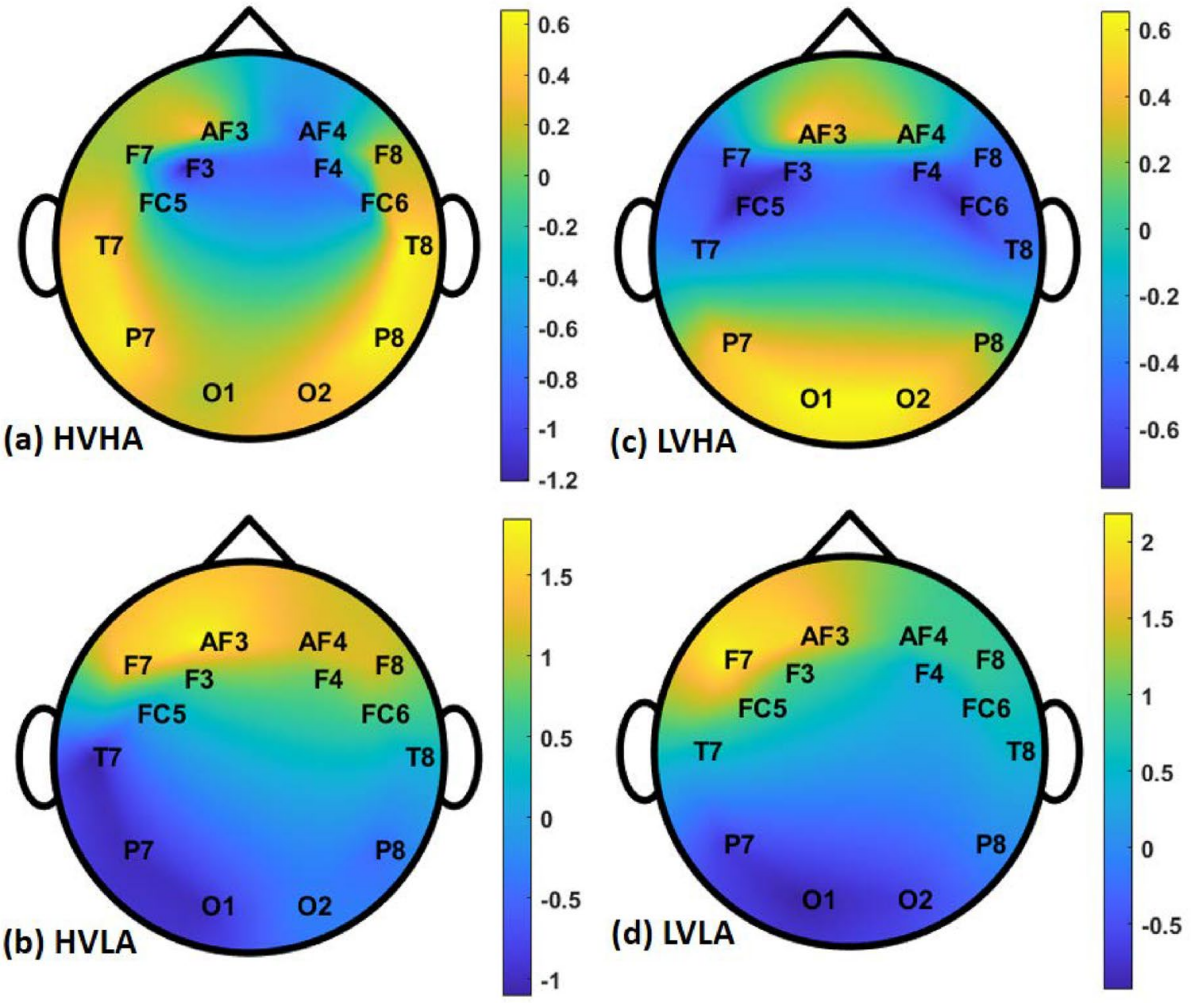
Interclass variation of EEG signals should be noticed for four emotion classes, the color bar represents the mean amplitude of 1-sec segment of EEG (**a**) Topological map of high valence high arousal (HVHA) class (**b**) Topological map of high valence low arousal (HVLA) class (**c**) Topological map of low valence high arousal (LVHA) class (**d**) Topological map of low valence low arousal (LVLA) class.

This dataset contains EEG signals from 69 participants, including 33 females and 36 males having written informed consent. The EEG system used for data collection was ANT Neuro, with eego^TM^sports model^31^. It has several features, including support for dry wearable EEG caps, wireless data streaming, and storage, a selectable sampling rate up to 2048 Hz, an 8-bit trigger input for ERP studies, 64 electrodes according to the international 10-20 electrode placement standard^31^. Based on the EEG-based emotion recognition literature review^37,38^ we follow most of the studies to incorporate only the 14 most significant channels for emotion recognition^39^ such as AF3, F7, F3, FC5, T7, P7, O1, O2, P8, T8, FC6, F4, F8, and AF4. The 1-sec segments of each of these 14 channels for the first participant are presented in Fig. 1. These 14 channels are mapped to topological structure for all the four labeled classes of HVHA, HVLA, LVHA, and LVLA as expressed in Fig. 2.

The age of the subjects varies from 20 to 56 years with a mean of 36.95 years. Sixteen different words were selected to show to the participants at different times to evoke memories. These words include excited, cheerful, bored, unhappy, disappointed, fearful, alert, aroused, idle, lively, calm, relaxed, pleased, still, dulled, and nervous. These emotion-denoting words were first described by^41^, showing the semantic similarity between induced emotions and the 28 affective words, from which 16 emotion-related words used in the proposed dataset were selected by^42^, and showed its significance to induce emotions in participants. The emotion-related worlds displayed to participants were previously used for fMRI-based and facial emotion recognition tasks in^21^ and^43^.

**Figure 3:**
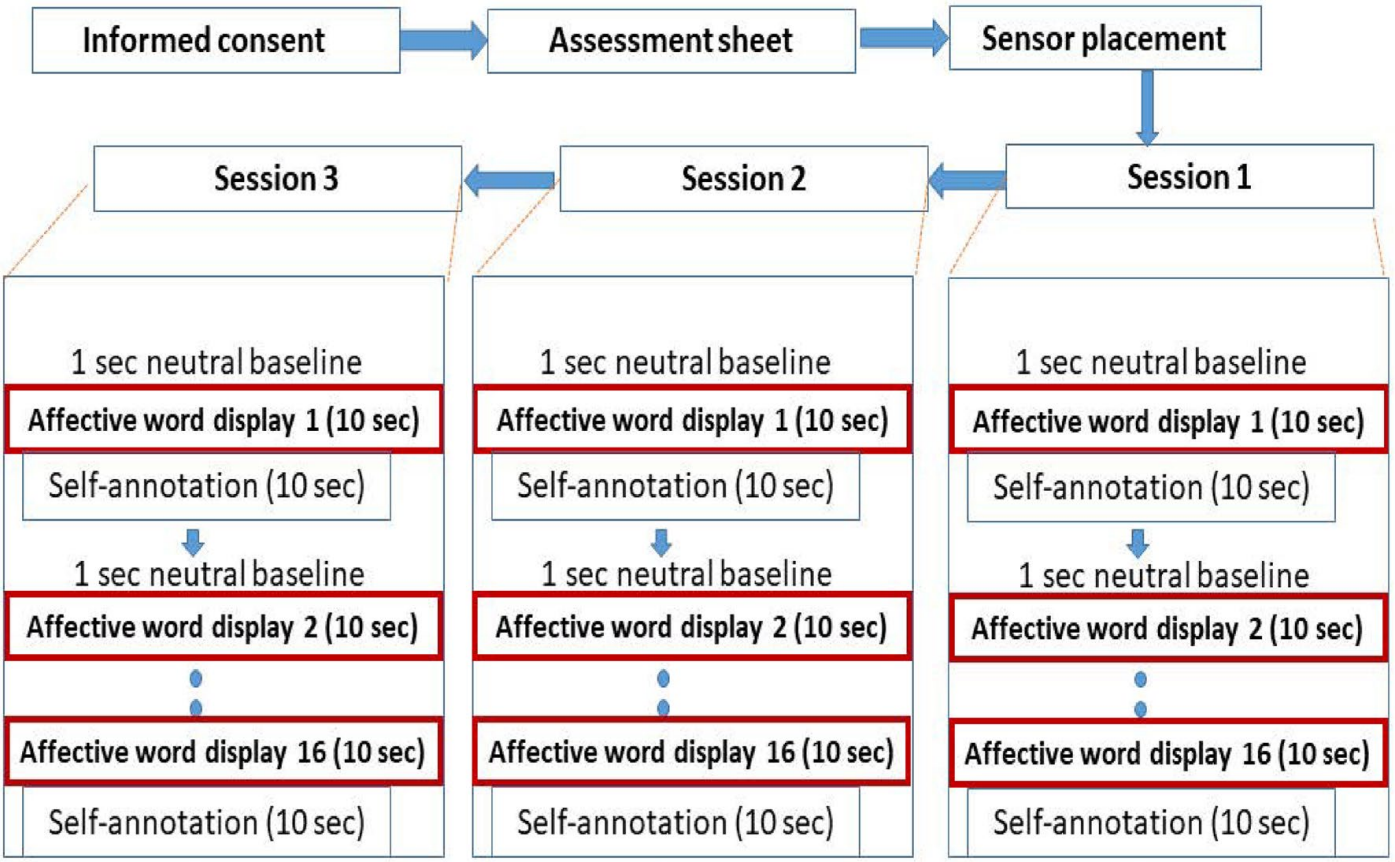
Overall experimental protocol for EEG data acquisition during emotional memory recall for 10-sec for each of the 16 affective words.

**Figure 4:**
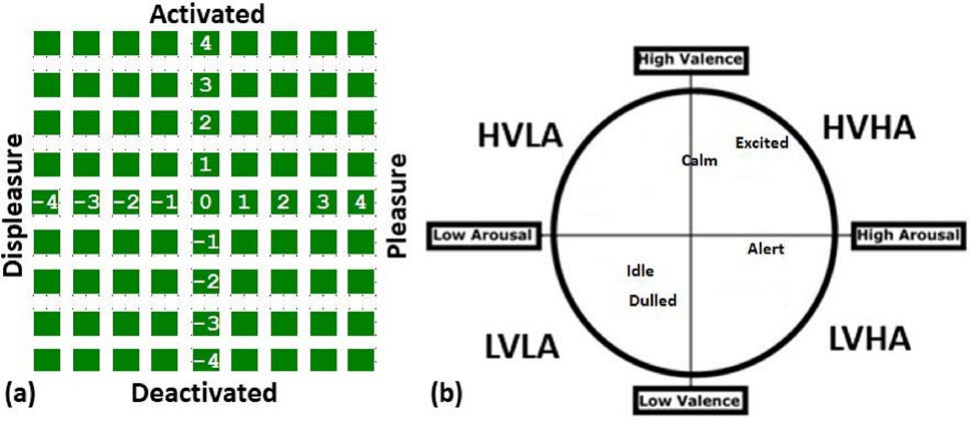
(**a**) The quadrant of valence and arousal (on the scale of $-4$ to 4) shown to each participant to select any single green box for self-annotation. (**b**) The selected box belongs to one of the quadrant such as high valence high arousal (HVHA), high valence low arousal (HVLA), low valence high arousal (LVHA), and low valence low arousal (LVLA) representing few examples of affective words with mean values.

There was a total of three sessions for each subject. Each session includes a presentation of these sixteen words in a random manner. Therefore, 48 words (16 words repeated randomly in three sessions) were presented to each subject, while EEG signals were recorded continuously during the whole experiment. The display of words is accompanied by event-related potentials (ERPs). The ERPs are used in this study for the sole purpose of getting the starting time of continuous EEG signal, where the activity of emotional memory begins. The 10-second EEG data after this ERP is segmented and used in the subsequent analysis. After each ERP, subjects were instructed to recall their memories for ten seconds relevant to the word displayed. The participants were also provided with explicit instructions to keep focus only on the memory of the displayed word to mitigate attention lapses. Therefore, the ten seconds of EEG data after each ERP was considered an emotional response to self-induced memories. Therefore, from continuous EEG signals, we have segmented a total of 480 s of EEG for 48 words ($48 \times 10$) for each subject. After ten seconds of display of each word, subjects were given another ten seconds to self-annotate their emotions felt during memory recall. The detailed description and timeline of the dataset acquisition protocol are presented in Fig. 3. The participants were briefed with the self-assessment manikins (SAM)^44^ to elaborate on the scale of valence (degree of positiveness or negativeness in emotion) and arousal (degree of feeling excited). Most of the publicly available datasets^25,26^ of EEG for emotion recognition incorporate the use of SAMs to visualize the scale of felt emotion. These SAMs are the standard pictorial representations used in the literature for the correct understanding of valence and arousal to the participants. Subjects can select values of valence (in the range of $-4$ to 4 from displeasure to pleasure) and values of arousal (in the range of $-4$ to 4 from deactivated to activated) from a 2D selection chart as shown in Fig. 4a. Figure 4b represents the mapping of a few examples of affective words with mean values to the four quadrants of valence and arousal such as high valence high arousal (HVHA), high valence low arousal (HVLA), low valence high arousal (LVHA), and low valence low arousal (LVLA).

**Table 3:** Relation between evoked words with self-annotation score of valence and arousal, and with four quadrants of HVHA, HVLA, LVHA, LVLA.

Word	Valence ($\mu$)	Valence ($\sigma$)	Arousal ($\mu$)	Arousal ($\sigma$)	HVHA	HVLA	LVHA	LVLA	None
Excited	2.8213	1.3409	2.942	1.3426	191	1	1	4	10
Cheerful	2.8357	1.4588	2.8454	1.3054	190	0	1	4	12
Bored	− 1.6763	1.7644	− 0.8019	2.191	22	14	50	109	12
Unhappy	− 2.3961	1.7003	− 0.942	2.4329	9	8	52	126	12
Disappointment	− 2.3382	1.8829	− 0.913	2.4834	10	8	59	118	12
Fearful	− 2.3623	1.6777	− 2.0628	2.6349	14	4	86	92	11
Alert	− 0.3671	2.3853	2.3092	1.6073	85	3	98	10	11
Aroused	1.7778	2.2008	2.0676	1.8787	147	9	24	13	14
Idle	− 1.0773	1.8018	− 0.4251	1.9738	50	12	39	85	21
Lively	2.5942	1.6219	2.8406	1.2766	183	0	7	3	14
Calm	2.3671	1.5361	1.1304	2.2027	150	37	5	4	11
Relaxed	2.6667	1.5201	1.5894	2.2856	161	30	5	4	7
Pleased	2.8841	1.5092	2.8019	1.4294	186	3	4	3	11
Still	− 1.2174	1.8424	− 0.7343	1.8518	36	20	36	98	17
Dulled	− 1.7536	1.6641	− 0.9034	1.941	18	8	41	125	15
Nervous	− 2.0676	1.7081	0.1787	2.611	19	6	91	81	10
Total	–	–	–	–	1471	163	599	879	200

$\mu$ represents mean and $\sigma$ represents standard deviation of score.

The distribution of valence and arousal is correlated with the emotional word displayed to the user. The detailed correlation of word-related emotional memories with a mean and standard deviation of arousal and valence is presented in Table 3. This Table also summarizes the relation between four classes of HVHA, HVLA, LVHA, and LVLA with evoked emotional memories. This study incorporates a wearable cap, which can be utilized in daily activities such as running, walking, cycling, reading, and high-intensity exercises. The purpose of using this device is to provide an emotion charting solution during any mobility and environment. Different brain regions are studied with this device for physical efforts^45^, but none of the studies is performed for emotion recognition with this ultra-mobile device. The variety of sizes covers the range of users with large, medium, small, child, infant, and baby. Another notable property of the acquired dataset is the wide range of age of participants and the total number of participants. The mean age of participants is 36.95 years, with a standard deviation of 9.67 years, which is remarkable compared to other competitive datasets expressed in Table 2.

**Figure 5:**
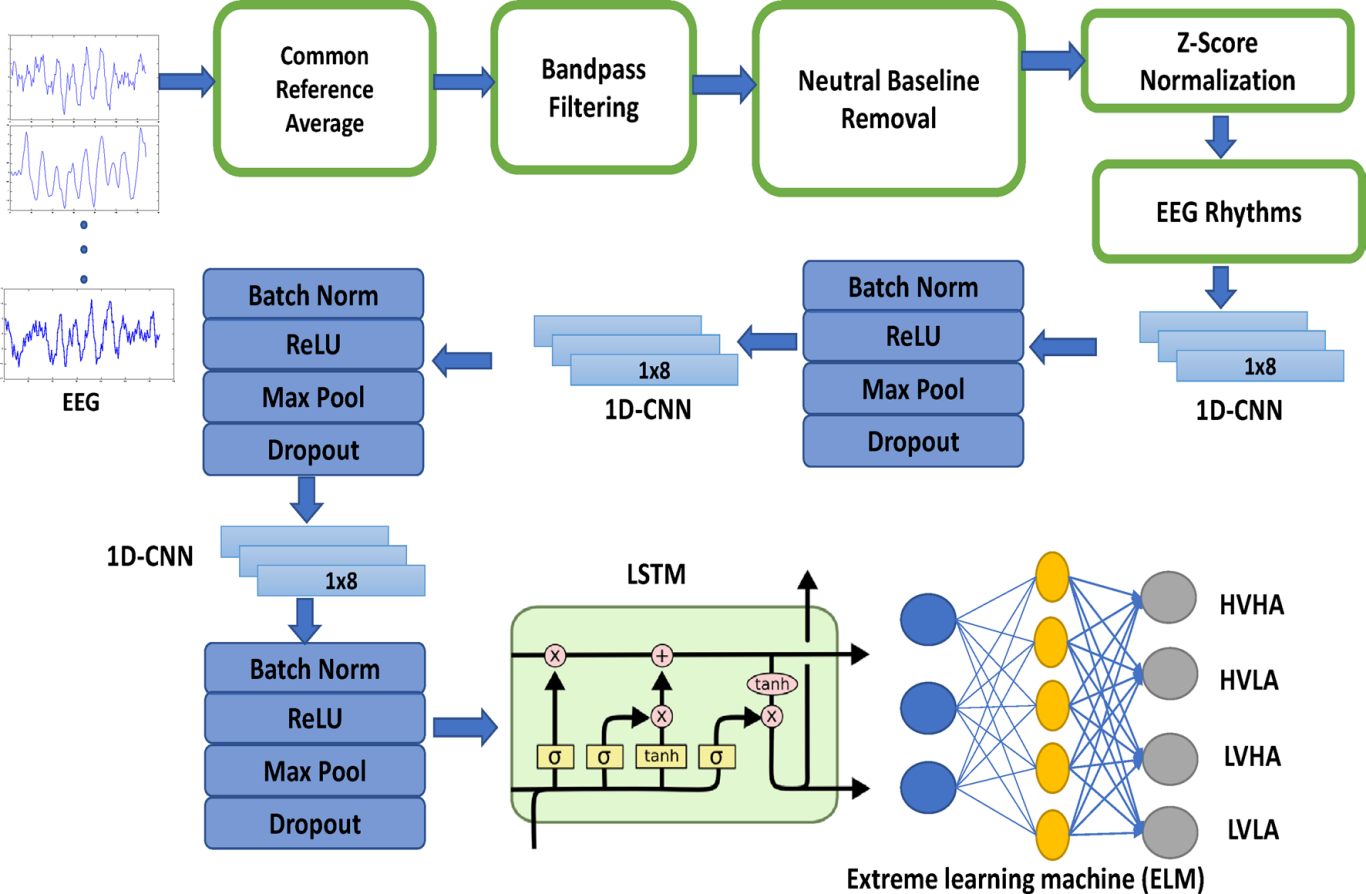
Block diagram of proposed methodology. CNN: Convolutional neural network, LSTM: Long Short-Term Memory.

### Methodology

The proposed methodology consists of pre-processing, feature extraction with 1D-CNN and LSTM, and classification using Extreme Learning Machine classifier. The complete block diagram of the proposed framework is presented in Fig. 5.

#### Pre-processing

A significant influencing factor for performance enhancement of emotion recognition includes proper de-noising of raw EEG signals. However, most research almost standardizes the processing 128 Hz frequency of physiological signals, enough for emotion recognition processes. Therefore, the EEG signals were downsampled to 128 Hz, while the common reference averaging is applied to raw EEG signals for standardization of all channels. The EEG is contaminated with low and high-frequency noises. Therefore, a passband filter of 1–50 Hz is applied for removing both the low frequency noises from body movements, and high frequency powerline interference from EEG signals.

**Figure 6:**
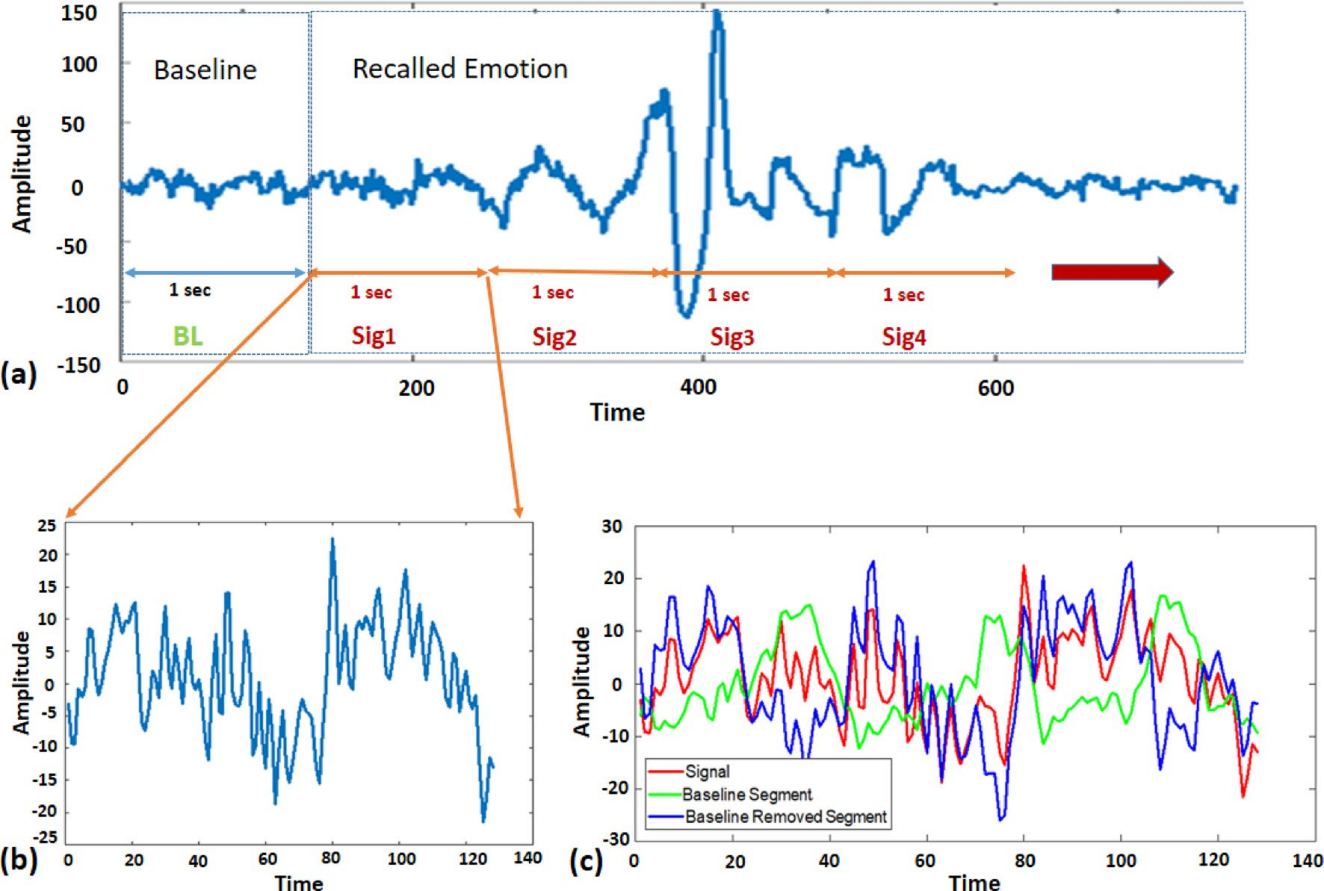
(**a**) EEG signal divided into 1 second neutral baseline segment, and 10 segments of 1 sec signal with evoked memory recalls (first four are displayed). (**b**) Each of the segment with recalled emotion selected (here first segment is selected) (**c**) Each of the selected segment is subtracted from baseline segment to highlight only the emotional content in the signal, results of segment 1 as signal, and baseline removed version is displayed.

EEG signals were continuously acquired from each subject while ERPs were recorded each time a word is displayed to them to evoke relevant emotional memory. The ten seconds of EEG data after each ERP is considered as a single sample with a unique label mapped on the valence and arousal scale. The ocular artifacts are removed by EEGLAB toolbox^46^. After filtering, all physiological signals are converted to 1-s segments. Similarly, the baseline signal is also subtracted from the signal recorded during memory recall. The baseline removal will result in a signal that only incorporate the memory recall-based emotional information. The 10-sec memory recall period is segmented into ten separate segments of 1-sec each. The 1-sec of data before the recall period is considered as the baseline, where there was no activity of memory recall. This baseline signal is subtracted from each of the ten segments of the memory recall period to remove the neutral baseline content and highlight the emotional response felt during memory recall as presented in Fig. 6. The signals are then standardized using z-score normalization to be ready for input to the deep neural network. In essence, the signals are enhanced by removing physiological artifacts, electrical interference, and baseline neutral activity. The EEG rhythms were extracted from the z-score normalized EEG signals using a Chebyshev type 2 filter with a stopband ripple of 10 dB. The resulting EEG rhythms were delta (1–4 Hz), theta (4–8 Hz), alpha (8–13 Hz), beta (13–30 Hz), and gamma (30–49 Hz).

#### One dimensional convolutional neural network

The primary reason to use the Convolutional Neural Network (CNN) feature detector from the physiological signal is parameter sharing. For instance, a 1x8 CNN filter with trained parameters can detect similar features in other parts of the signal and other channels as well. For EEG signals, the input to the 1D-CNN is $14 \times 128$. There are 14 channels of EEG and each channel contains 128 values of 1-s. Compared to 2D-CNN, one-dimensional convolutional neural networks offer distinct advantages for sequential and time-series data, such as temporal signals or sequences. As physiological signals are continuous, long-term repetitive patterns, a 1D convolutional feature detector can make use of parameter sharing. This is because features learned in one part of the signal are useful for other parts of the signal as well. At the same time, the parameters learned from one channel can be useful for other channels as well. This phenomenon can be explained by the internal representation of convolutional features.

**Figure 7:**
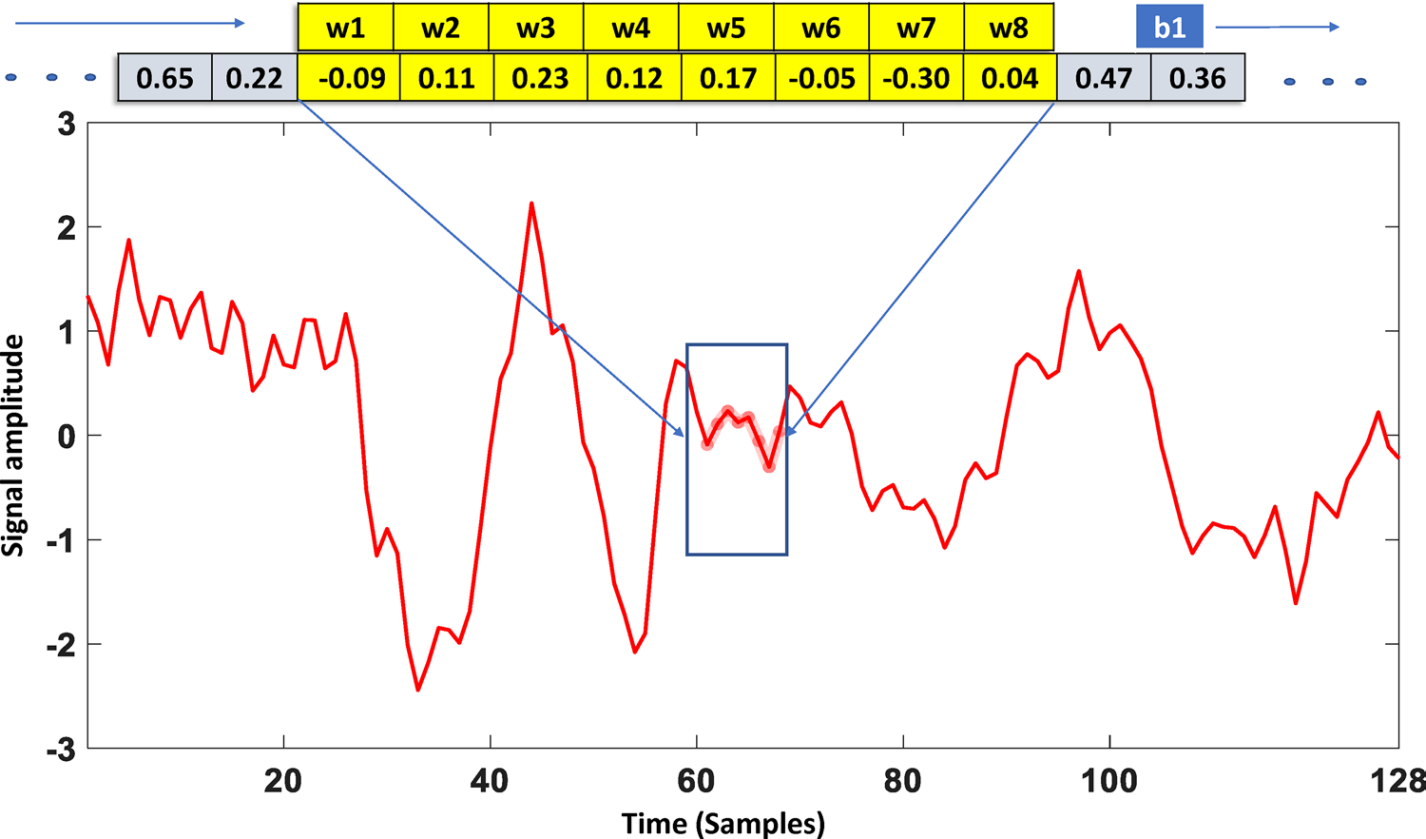
1D Convolutional kernel applied to sample index 61–68 (values highlighted in yellow color, convolved with the kernel weights) of AF3 channel of EEG signal. w1 to w8 represents eight weights of convolutional kernel, while b1 represents bias of kernel.

**Figure 8:**
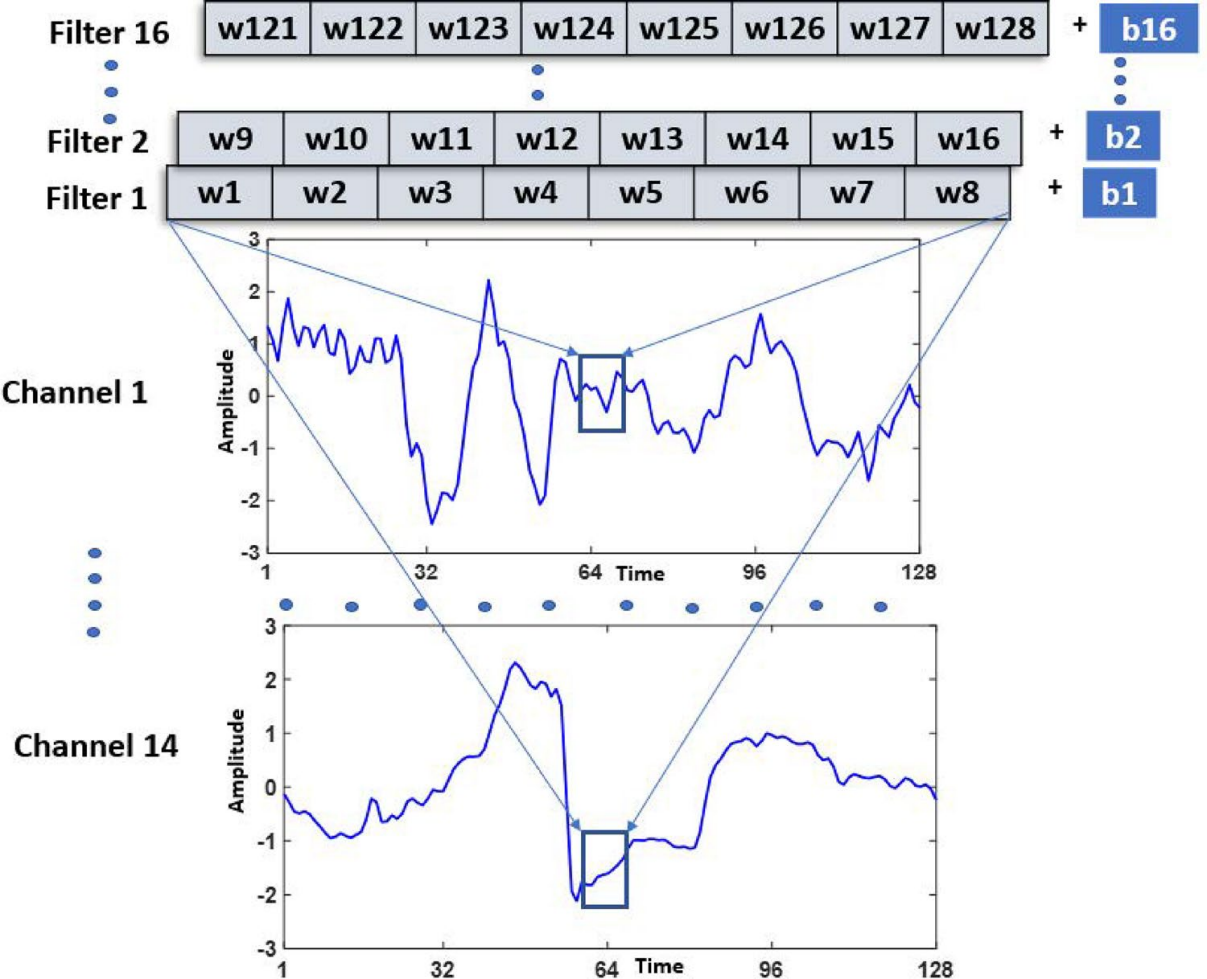
All the 16 1D convolutional kernels, applied on 14 channels of EEG Signal.

Figure 7 represent a 1-s preprocessed segment of one of the fourteen channel, known as AF3. Similarly, a vector of 1 x 8 is represented as a kernel of 1D-CNN. The values of the kernel are $w_{1}, w_{2},..., w_{k}$, where *k* represent size of the kernel. These eight values of the kernel are known as learnable parameters or weights. For filter 1, these values are represented as $w_{1}, w_{2},..., w_{8}$. There are a total of 16 filters (f(1),f(2),...,f(16)) in the first layer of 1D-CNN, and each filter contains these eight parameters. The number of 16 CNN filters was empirically selected with preliminary experiments. The reduction of CNN filters to 8 or 4 resulted in less performance due to a lack of representational capacity or an inability to extract useful repetitive patterns from EEG signals. Similarly, increasing filters to 32 or 64 does not improve the classification accuracy at the cost of additional computational complexity. Each filter also has one bias value in addition to these weights. Therefore, there are 16 (filters) x 9 (filter weights + bias) =144 learnable parameters of the first layer of 1D-CNN. $z_{1}, z_{2},..., z_{v}$ represent preprocessed signal after z-score normalization, where v is the length of the 1-sec signal with 128 values. Therefore, Fig. 7 represents, one of the 16 kernels (f(1)), convolved with one of the 14 EEG channels. The result of this convolution is presented in Eq. (1).

1 $$\begin{aligned} f(1)_{v} = b_{1} + \sum _{i=1}^{k} \left( w_{i}*z_{v + i} \right) \end{aligned}$$

The size of the output of 1D-CNN for each of the signal channels is kept the same as the input to the 1D-CNN, which is 1 $\times$ 128 vector. To accomplish this, padding of the signal is required. Without padding ($p=0$) the output length of 1D-CNN will be computed to 121, as given in Eq. (2). sl represent stride length, which is selected to $1 \times 1$. v is 128, k is 8, therefore, $((128+0-8)/1)+1=121$. The visualization of strides of 1D-convolutions can be presented in Fig. 7. In Fig. 7, the f(1) is convolving with signal z, with index values (v) from 61 to 68. The next convolution operation will be performed after applying a stride of 1 $\times$ 1, therefore, the f(1) is convolving with the index values (v) of z from 62 to 69.

2 $$\begin{aligned} a = \frac{v+p-k}{sl} + 1 \end{aligned}$$

p represents the size of padding, and for the same output length, we should apply padding to the signal. The p can be calculated using $p = k-1$. Therefore, we should pad the original signal with 7 values. We applied, zero padding, therefore, after the signal is padded with seven zero values, the (2) gives the output length of size 128. It is important to note that, the same filter f(1) will be convolved with the other thirteen channels of EEG signals to extract features. This mechanism of the f(1) as well as other kernels applied to each of the 14 channels is presented in Fig. 8. For each of the channels, the result of the convolutions will be a 1 $\times$ 128 vector. Therefore, the size of activations computed by f(1) is 14 $\times$ 128. We just need to train the bias and weights of the f(1) kernel using the back propagation technique for the classification of a specific emotion.

There are 16 different kernels with parameters and bias terms presented in Eqs. (3) and (4), and so on for other layers. Each of the 16 kernels computes 14 $\times$ 128 activations after the first layer of 1D-CNN. Therefore, the total number of activations after layer 1 of 1D-CNN is 14 $\times$ 128 $\times$ 16. The visual representation of these 16 kernels applied to 14 channels of 128 values is provided in Fig. 8.

3 $$\begin{aligned} f(2)_{v}= & {} b_{2} + \sum _{i=9}^{8+k} \left( w_{i}*z_{v + i} \right) \end{aligned}$$

4 $$\begin{aligned} f(16)_{v}= & {} b_{16} + \sum _{i=121}^{8+k} \left( w_{i}*z_{v + i} \right) \end{aligned}$$

#### Batch normalization

The input data of the neural network needs to be normalized, to accelerate the learning of parameters, by optimizing steps of gradient descent. The same phenomenon is critical in the hidden layers of deep neural networks. For instance, the normalization of activations of hidden layer 1, will result in efficient learning of parameters in hidden layer 2 and so on. Therefore batch normalization of activations of layer l, can improve the learning efficiency of parameters between layer l and layer l + 1. In deep learning literature, the common practice is to apply batch normalization after computing summations, such as $f(1)_{v}, f(2)_{v},...,f(16)_{v}$ before applying activation function. Therefore, we use the same standard and applied batch normalization after computing $f(1)_{v}, f(2)_{v},...,f(16)_{v}$ and before applying ReLU activations. This normalization will be performed by Eq. (5), and so on to other fifteen kernels to compute normalizes values such as $nf(1)_{v}, nf(2)_{v},...,nf(16)_{v}$. This equation can be written in a generalized form as shown in Eq. (6), where q represents the number of kernels varying from 1 to 16. However, we do not want these values with zero means and variance equal to one. We just want to standardize the mean and variance of these values. For that purpose, batch normalization adds two learning parameters of $\gamma$ and $\beta$ for each of the 16 kernels. This can be represented in generalized form in Eq. (7). Therefore, the total number of learning parameters for batch normalization is 16 $\times$ 2 = 32 parameters. However, the size of activations will remain the same as in the previous layer.

5 $$\begin{aligned} nf(1)_{v}= & {} (f(1)_{v}-\mu )/\sigma ^{2} \end{aligned}$$

6 $$\begin{aligned} nf(q)_{v}= & {} (f(q)_{v}-\mu )/\sigma ^{2} \end{aligned}$$

7 $$\begin{aligned} bnf(q)_{v}= & {} \gamma (q) * nf(q)_{v} + \beta (q) \end{aligned}$$

#### Activation function

There are various activation functions that can be used in deep neural networks. It includes sigmoid function, tanh function, and ReLU (rectified linear unit) activation function. The mathematical expressions of these activation functions are presented in Eqs. (8), (10), and (10) respectively.

8 $$\begin{aligned} a_{S}= & {} \frac{1}{1+e^{-(bnf(q))}} \end{aligned}$$

9 $$\begin{aligned} a_{T}= & {} \frac{e^{bnf(q)}-e^{-bnf(q)}}{e^{bnf(q)}+e^{-bnf(q)}} \end{aligned}$$

10 $$\begin{aligned} a_{R}= & {} max(0,bnf(q)) \end{aligned}$$

The sigmoid function is normally used in only the last layers of the deep neural network. Tanh function is a shifted version of the sigmoid and is always a better choice compared to the sigmoid function. This is because input data to DNN is normalized for zero means, which can easily be incorporated into tanh function. We have used sigmoid and tanh activation functions in recurrent layers. However, the drawback of both of these functions for CNN layers is their tendency to output values close to zero when the input values are large. This will result in the deceleration of gradient descent learning. In contrast, the ReLU activation is a more suitable choice, as it calculates the derivative to be zero for negative input, and the derivative to be one for positive input values. We have incorporated ReLU function to get the output of $a_{R}$ as shown in Eq. (10. It is also trivial to consider that we are using a non-linear activation function because of the complex and non-linear distribution of our multi-class emotions data. Also, there is no learnable parameter involved in computing activation values and the size of activations remains the same as in the previous layer.

#### Max pooling layer

The pooling layer minimize number of features and hence avoid the problem of over-fitting. We incorporated max pooling by setting two hyper-parameters such as stride and filter size. The filter size is set to be 1 $\times$ 2, while the stride is set to 1 $\times$ 1. The padding is used with zero value for the last value of the signal, just to fit the kernel. For one dimensional data with a short size kernel and stride length of sl, the max-pooling does not change the dimensions of output features. The dimension of the output layer can be computed similarly to the convolutional layer by using Eq. 2. In the absence of any padding, the output dimension will be $((128+0-2)/1)+1=127$. Therefore, we only need to pad ($p=1$) the last value with zero to make the output length equal to the input. However, the main objective of using max pooling for 1D signals is to enhance the generalization property of the extracted features and hence avoid overfitting. The output values of max pooling with given parameters can simply be computed from Eq. (11. Here, $a_{R,v}$ represents the vth value of the feature after applying the ReLU activation function, and $mp_{v}$ represents the vth value of the feature after applying max-pooling of stride one and kernel size 2.

11 $$\begin{aligned} mp_{v} = max(a_{R,v},a_{R,v+1}) \end{aligned}$$

#### Dropout layer

The dropout layer is added to randomly discard features from the current layer. The probability of dropout is selected to be 0.5, therefore, 50% of neurons and their corresponding weights will be deactivated. It is critical to consider that size of output activations will not be changed, but for every dropout layer with a probability of 0.5, half of the neurons will be shut off. The dropout will generate a vector of random numbers, with half of the values of the total neuron in the current hidden layer, and then discard those randomly selected neurons. The random selection is based on the fact that we do not want to rely on any feature in order to generalization the performance of neural and thus avoid overfitting.

#### Long short-term memory

Long short-term memory (LSTM) is a particular type of recurrent network to conquer the long-term dependency in RNN. The long short-term memory layer is incorporated to extract both short and long-term repetitive pattern-based features. The output of previous 1D-CNN layers is 14 $\times$ 128 $\times$ 16, which is then flattened to a vector of size 1 $\times$ 28,672. This is quite a long sequence input, which is difficult to learn from standardized backpropagation through time resulting in a vanishing gradient. The gated cells and memory added to LSTM solve these problems. Therefore, the 28,672-sized vector is passed as input to the LSTM layer with 32 neurons. The total learnable parameters of LSTM layer are (128 $\times$ 28,672) + (128 $\times$ 32) + 128 = 3,674,240. The sigmoid is used as a gate activation function while tanh is used as a state activation function. The input weight of LSTM is initialized using the glorot scheme with a small Gaussian value and mean zero. To avoid exploding or vanishing gradients, the recurrent weights were initialized using an orthogonal scheme. Unit forget gate bias initializer is incorporated to achieve better performance with one-dimensional signals.

#### Extreme learning machine

The extreme learning machine^47^ is a single hidden layer feed-forward neural network with strong generalization capability without iterative tuning. Unlike Artificial Neural Networks, the ELM does not require tuning and periodically assigns hidden neurons. It randomly chooses biases and input weights of hidden layers and determines the output weights using least squares methods, resulting in the low computational time of ELM^48^. Literature shows that ELM performs better than SVM with CNN-extracted features^49^. Therefore the proposed framework is improved with the addition of an extreme learning machine that empirically performs better than fully connected layers and an SVM classifier for emotion recognition. ELM uses layered architecture for fast computations and shows promising results in recognizing EEG-based emotions^50^. Extracted features from the 1D-CRNN fed to ELM for classification. The number of hidden neurons selected to train the ELM classifier is 9000. The training samples were further divided into 80:20 of training data and validation data, used to train and validate the ELM classifier.

**Figure 9:**
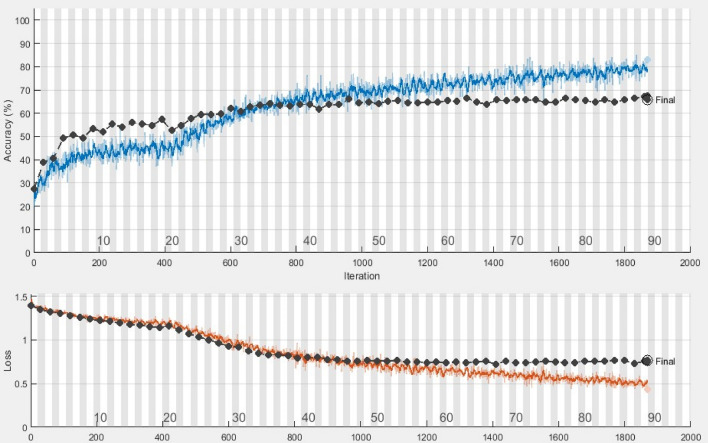
Epoch wise training and validation score. Black line represent validation accuracy and validation loss, blue line represent training accuracy, and red line represent training loss.

#### Performance evaluation

Performance evaluation and comparative analysis is performed for each of the EEG rhythm using precision, sensitivity, specificity, and F-measure. The computation of precision is presented in Eq. (12). It compute the closeness or dispersion of measurement of various classes. Similarly, the performance of our model is also established for sensitivity, specificity, and F-measure as measured by Eq. (13, 14, and 15.

12 $$\begin{aligned} Precision= & {} \frac{TP}{TP + FP} \end{aligned}$$

13 $$\begin{aligned} Sensitivity= & {} \frac{TP}{TP + FN} \end{aligned}$$

14 $$\begin{aligned} Specificity= & {} \frac{TN}{TN + FP} \end{aligned}$$

15 $$\begin{aligned} F-measure= & {} \frac{2 * Precision * Sensitivity}{Precision + Sensitivity} \end{aligned}$$

## Results and discussion

The experimentation protocol involved the combination of digital signal processing and deep neural networks. The training of the neural network is performed on a Core-i5 machine for 90 epochs. The batch size is selected to be 240, with an initial learning rate of 10E-3. The gradient decay factor of 0.99 is used with the ADAM optimizer as training parameters. The training of the proposed framework is presented in Fig. 9. The samples in four classes have a large difference and thus create the problem of imbalanced classes. The HVLA is the class with the least number of 163 samples. After the segmentation step of preprocessing (divide each 10-s segment into ten separate 1-sec segments), the samples of each class become 10-fold. Therefore, the HVLA class has a minimum of 1630 samples of 1-s each. To avoid the imbalanced class problem, we randomly discarded additional samples above 1630 from each class. Therefore, the total number of samples used for experimentation purposes was 1630 $\times$ 4 = 6520. The dataset is randomly divided into train test ratios of 80 and 20 percent respectively. This random split is applied three times, and the average results are presented for these three random splits. In EEG-based emotion recognition research, there is not a single standard for the selection of k-value in cross-validation, and the 5-fold, 10-fold, and 15-fold are associated with potential risks based on the randomness of data split when dealing with a small dataset, or poor generalization to unseen data^19^. The selection of the k-value in cross-validation is significant for ensuring the generalizability of the model and therefore, this study further investigates the performance with a more rigorous approach by leaving one subject out of validation.

**Figure 10:**
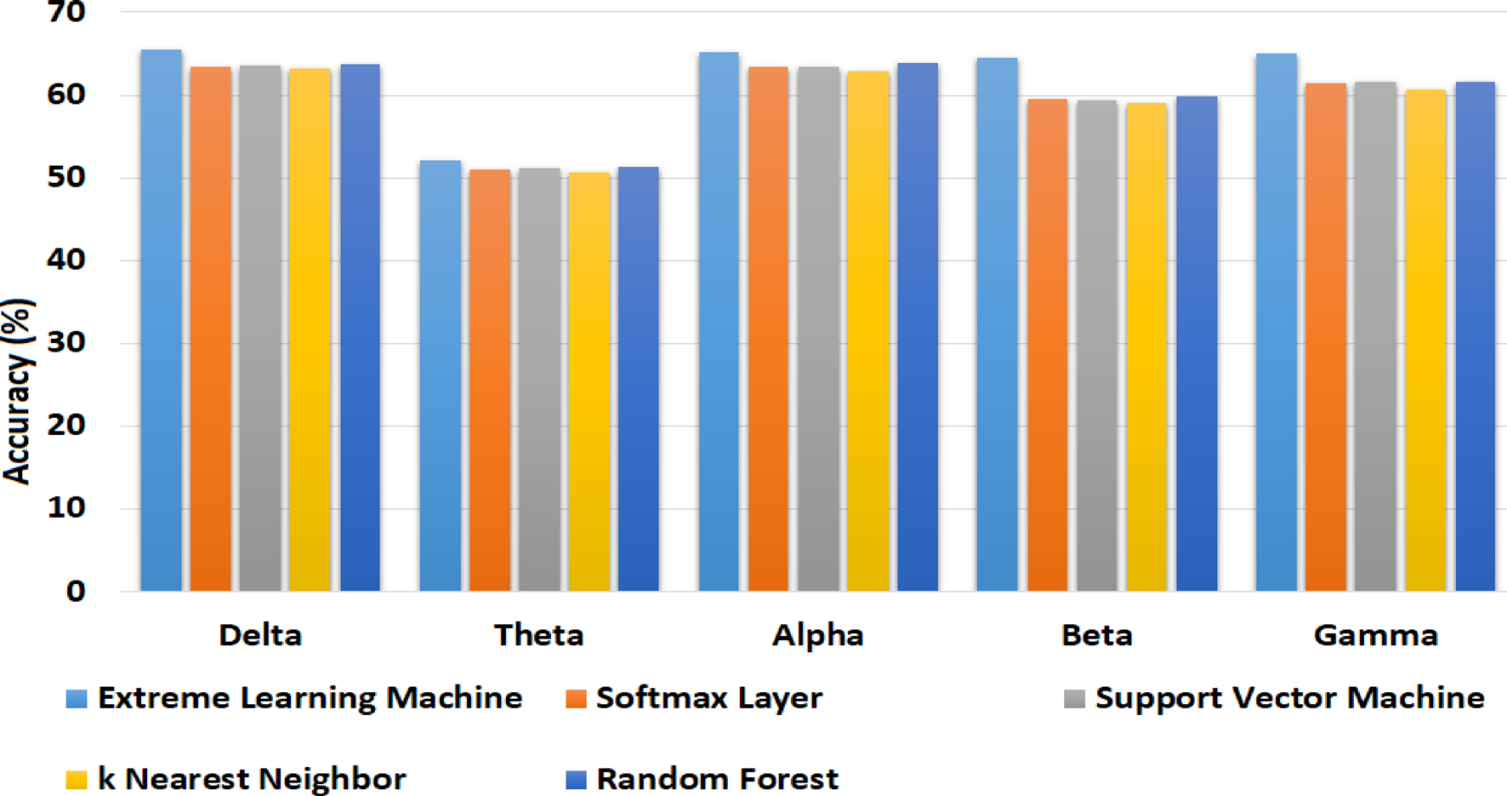
The accuracy of all EEG rhythms, after feature extraction with 1D-CRNN, and compared with Extreme Learning Machine, Support Vector Machine, k Nearest Neighbors and Random Forest Classifiers, showing the significance of the use of ELM afer 1D-CRNN.

**Table 4:** Confusion matrix of EEG combination of all bands.

Output class	Target class				Total
	HVHA	HVLA	LVHA	LVLA	
HVHA	202 $15.5\%$	23 $1.8\%$	82 $6.3\%$	104 $8.0\%$	$49.1\%$
HVLA	12 $0.9\%$	270 $20.7\%$	15 $1.2\%$	9 $0.7\%$	$88.2\%$
LVHA	13 $1.0\%$	24 $1.8\%$	202 $15.5\%$	31 $2.4\%$	$74.8\%$
LVLA	99 $7.6\%$	9 $0.7\%$	27 $2.1\%$	182 $14.0\%$	$57.4\%$
Total	$62.0\%$	$82.8\%$	$62.0\%$	$55.8\%$	$65.6\%$

The boxes shows both the number of samples and the percentage of samples to the total number of samples. Similarly, total percentages below the the matrix shows the percentage of true positive rate from that specific class, and right side of the matrix with percentages of precision, the total percentage value at right bottom shows the overall accuracy of model.

**Figure 11:**
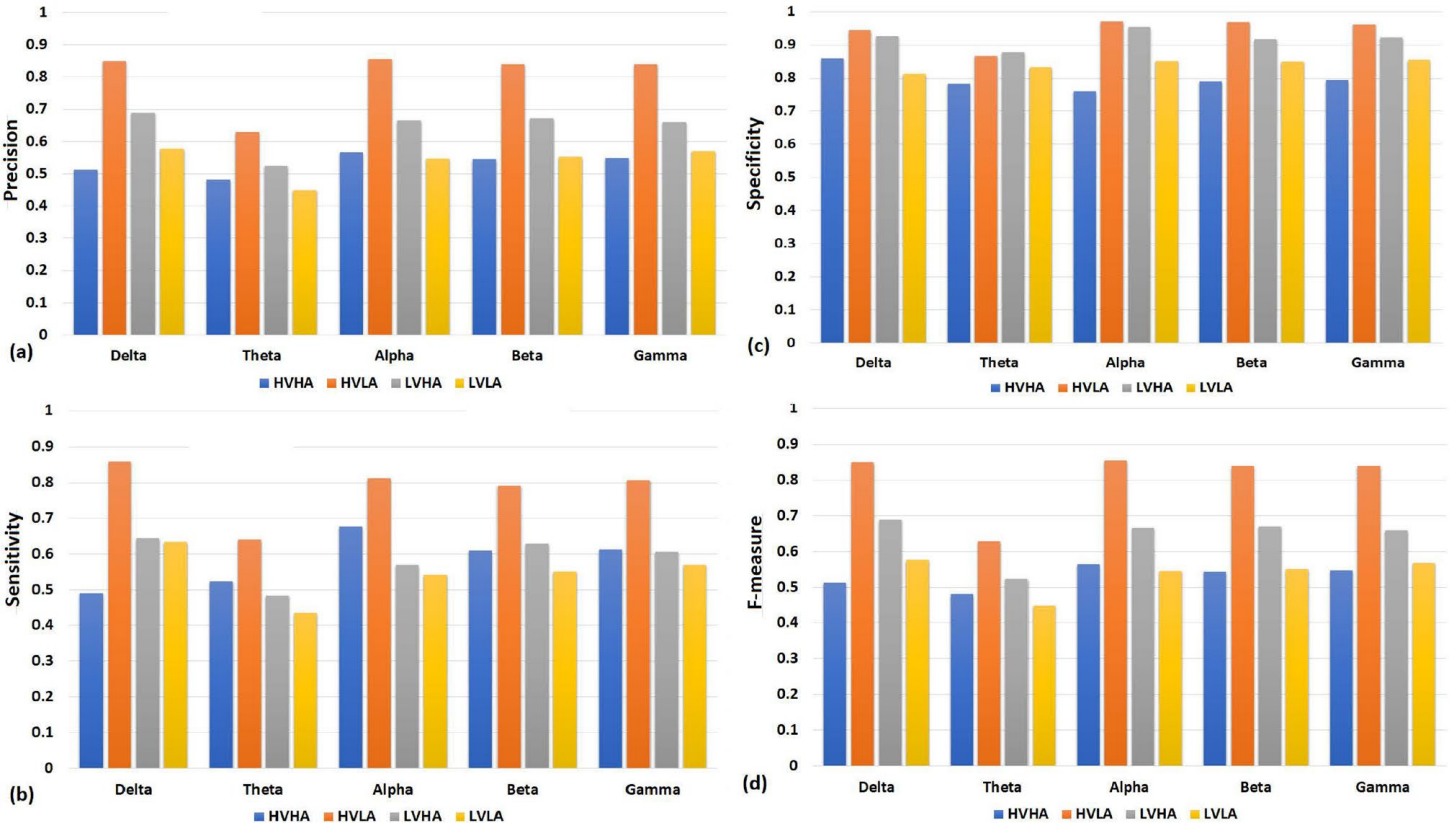
The performance evaluation of all EEG rhythms against HVHA, HVLA, LVHA, and LVLA. In general, HVLA class has the highest precision, sensitivity, specificity, and F-measure values. (**a**) In precision analysis, alpha frequency band performs better compared to other frequency bands. (**b**) In sensitivity analysis, delta frequency band generally has higher sensitivity. (**c**) In specificity analysis, alpha frequency band generally has higher specificity. (**d**) In F-measure analysis, delta frequency band generally has higher specificity compared to other frequency bands.

The results are computed by dividing the EEG signals into delta (1–4 Hz), theta (4–8 Hz), alpha (8–13 Hz), beta (13–30 Hz), and gamma (30–49 Hz) bands. These five EEG bands were created by applying chebyshev type 2 filter on EEG signals with stopband ripple of 10dB. Delta band is dominant in sleep stages, while theta band occurs during deeply relaxed, tiredness and drowsiness states. Alpha rhythms occur during the passive attention state, while beta rhythms occur during anxiety and active state of mind. The gamma rhythms usually occur during concentration and problem-solving. The division of EEG signals into frequency bands helps feature extraction related to specific mental states.

The average accuracy for the four-class classification of HVHA, HVLA, LVHA, and LVLA is 65.5%, 52.1%, 65.1%, 64.6%, and 65.0% for delta, theta, alpha, beta, and gamma rhythms respectively. The results comparison of a softmax layer with an Extreme learning machine is provided in Fig. 10 of the revised manuscript. The empirical results obtained with this comparison show the significance of ELM compared with softmax layer and other classifiers such as Support Vector Machine, k Nearest Neighbors, and Random Forest. By combining these bands with the use of ELM, the accuracy is 65.6% for four-class classification. The detailed results with a combination of these bands are presented as a confusion matrix in Table 4.

It is significant to perform both class-wise and EEG rhythm-wise performance analysis to get the better insight into performance of various parameters involved in this study. Figure 11 shows that HVLA class performs better compared to HVHA, LVHA, and LVLA for all the EEG rhythms. We have removed the class imbalance problem by removing random samples from each class. The performance of HVLA class is better as it has fewer samples, and none of the samples of HVLA were removed during class balance.

The classwise precision results of all of these five rhythms are presented in Fig. 11a. The theta rhythm performs less than other rhythms for all four classes of emotions. The specificity of each class for all the rhythms is higher compared to sensitivity or recall except HVLA class. Similarly, higher recall or sensitivity is measured for HVHA class except for the delta rhythm, where recall of LVLA class is higher. This exception can also be observed in higher specificity of HVHA for delta rhythm compared to other EEG rhythms as presented in Fig. 11b,c. A similar behavior of HVHA class can be observed in F-measure as shown in Fig. 11d.

Memory-induced emotion recognition is an emerging area and there are very limited studies that incorporate the scenarios close to the real-world environment. For instance^22^, achieves 63% of accuracy for the three-class classification of positive emotion, negative emotion, and neutral. The subjects were shown personalized images and asked to recall memories associated with those images. Temporal frequency features were extracted and passed through a linear discriminant analysis (LDA) classifier for recognition of two emotion classes and a neutral state. Another study^23^ incorporated discrete wavelet transform (DWT) based feature extraction, principal component analysis for selection of features, and support vector machine (SVM) for the classification of binary classification of feeling disgusted or not. The subjects were asked to remember unpleasant odors and self-annotate whether they feel disgusted or not. They achieved 90% accuracy for the presence or absence of single emotion of disgust. In a recent study^24^, subjects were shown stimulus videos, and after the video stopped, they were asked to close their eyes and remember the recently viewed stimulus videos while acquiring their EEG signals. They achieved an accuracy of 54.52% for six basic emotions of happy, sad, fear, anger, surprise, and disgust.

**Table 5:** Comparison of proposed methodology with state-of-the-art techniques with memory-induced emotion recognition dataset using EEG signals.

Technique	Temporal and frequency domain features with Linear discriminant analysis (LDA) classifier^22^	Wavelet transform feature extraction, Principal component analysis for feature selection, and SVM for classification^23^	Differential entropy features, and SVM for classification^24^	1D-CRNN-ELM (Proposed)
First random split (%)	52.60	60.58	60.04	65.64
Second random split (%)	53.83	61.12	59.43	66.03
Third random split (%)	54.22	60.97	59.35	65.26
Mean accuracy (%)	53.54	60.89	59.61	65.64
Standard deviation (%)	0.69	0.23	0.31	0.31

Table 5 represents a detailed comparison of the proposed methodology with existing techniques of memory-induced emotion recognition. The results from 69 participants with only 14 EEG channels exhibit the generalization of the proposed model. Words were displayed to evoke emotional memories in the participants, which can induce subjective memories compared to the odors, images, and stimulus videos based on short-time memory recalls. The proposed methodology outperforms conventional machine learning techniques for four emotion classes, fewer EEG channels, a large population size, and evoked words for more subjective emotional memory recall to mimic the real-world environment.

**Figure 12:**
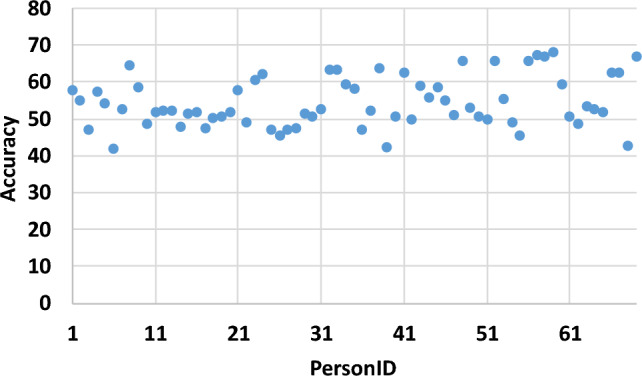
Leave one subject out validation results for all the 69 participants of this study with mean = 54.51 and standard deviation = 6.77.

The leave one subject out validation strategy is used to further investigate the validation of proposed methodology and user independence. The extensive experimentation is performed with 69 subjects, while the proposed model is trained on 68 subjects and tested on one of the subjects with unseen data. This is repeated for all the 69 subjects and accuracy of four class classification results are obtained and presented in Fig. 12. This figure shows the scatter plot of percentage accuracy against each of the 69 subjects tested with unseen data. The multi-class classification accuracy for HVHA, HVLA, LVHA, and LVLA classes are obtained with mean percentage accuracy of 54.51 and standard deviation of 6.77. The leave one subject out results are significantly lower than random splitting of data, because of the individual differences of EEG signals. Most of the studies incorporated limited number of participants, making it difficult to generalize the findings to a larger population. Therefore, these results suggests the use of other techniques to minimize the sensitivity of a model to inter-subject variability such as contrastive learning^51^ in the future studies.

The advantages of the proposed combination of 1D-CRNN-ELM include its inherent property of temporal and sequential for analyzing EEG data. It captures both short-term and long-term temporal dependencies in EEG signals for improved recognition of challenging memory-induced emotions. Combining 1D-CNN and LSTM is significant, because CNN layers facilitate spatial feature extraction, while LSTM enables complex temporal patterns related to memory-induced emotions. Similarly, ELM helps in the efficient training of spatiotemporal features extracted by CNN and LSTM layers. These advantages help in achieving better emotion recognition performance compared to state-of-the-art techniques for the same dataset of memory-induced emotions. However, the proposed framework has the disadvantage of increasing the complexity of the model and less generalization as seen with the leave one subject out validation results. The less generalization of the model is observed as around 10% less mean accuracy by leaving one subject out of validation compared to the mean of three random splits. The results of this study are encouraging by supporting our hypothesis that the deep learning model combining both CNN and LSTM can improve emotion recognition performance for challenging memory-induced emotions mimicking real-world scenarios. The dataset is challenging because of subjective memory recalls based on minimal evoking affective words, and the person can lose concentration while recalling emotional memories can contribute to less emotion recognition performance as anticipated with a complex deep learning framework. However, the performance can be improved with much more sophisticated deep learning algorithms in future work, and with the addition of other modalities such as ECG signals.

## Conclusions

This study proposed a deep learning technique for improving memory-induced emotion recognition performance and constructed a dataset of EEG signals acquired during highly subjective emotional memory recall. Affective words were randomly displayed to participants in three sessions to think about emotional memory for ten seconds. The data acquisition is performed with an ultra-portable, wearable cap sensor from 69 subjects with self-annotation on the dimensional scale of valence and arousal. The significance of the dataset is explored with the proposed framework of 1D-CRNN feature extractor with ELM classifier used to recognize four classes of dimensional emotion models known as HVHA, HVLA, LVHA, and LVLA. The proposed algorithm achieved a mean accuracy of 65.64% for four class classifications, better than state-of-the-art techniques used for memory-induced emotion recognition for the same dataset. The benchmark results with five EEG rhythms and their combination show the effectiveness of the proposed deep learning technique for memory-induced affect recognition evoked with affective words. The limitation of the acquired EEG dataset is the number of emotion classes and only EEG modality. Future work can incorporate more emotion classes and ECG modality with memory-induced emotion recognition. It would be of considerable interest to know what changes from baseline in spectral features indicate valence and arousal values with contrastive learning methods to overcome intra-subject variability in future work. This research will provide a baseline for researchers to develop emotion recognition algorithms in less constrained, real-world environments as recalling autobiographical memories during daily activities.

## Data Availability

The datasets used and/or analysed during the current study available from the corresponding author on reasonable request.
